# Advanced reactivity-based sequencing methods for mRNA epitranscriptome profiling†

**DOI:** 10.1039/d4cb00215f

**Published:** 2024-12-10

**Authors:** Zhihe Cai, Peizhe Song, Kemiao Yu, Guifang Jia

**Affiliations:** a Synthetic and Functional Biomolecules Center, Beijing National Laboratory for Molecular Sciences, Key Laboratory of Bioorganic Chemistry and Molecular Engineering of Ministry of Education, College of Chemistry and Molecular Engineering, Peking University Beijing 100871 China guifangjia@pku.edu.cn; b Peking-Tsinghua Center for Life Sciences, Peking University Beijing 100871 China; c Beijing Advanced Center of RNA Biology, Peking University Beijing 100871 China

## Abstract

Currently, over 170 chemical modifications identified in RNA introduce an additional regulatory attribute to gene expression, known as the epitranscriptome. The development of detection methods to pinpoint the location and quantify these dynamic and reversible modifications has significantly expanded our understanding of their roles. This review goes deep into the latest progress in enzyme- and chemical-assisted sequencing methods, highlighting the opportunities presented by these reactivity-based techniques for detailed characterization of RNA modifications. Our survey provides a deeper understanding of the function and biological roles of RNA modification.

## Abstract 2

This review explores the latest progress in reactivity-based sequencing methods for nine key mRNA modifications, focusing on their potential features for precise and sensitive mapping and quantification.

## Graphical abstract

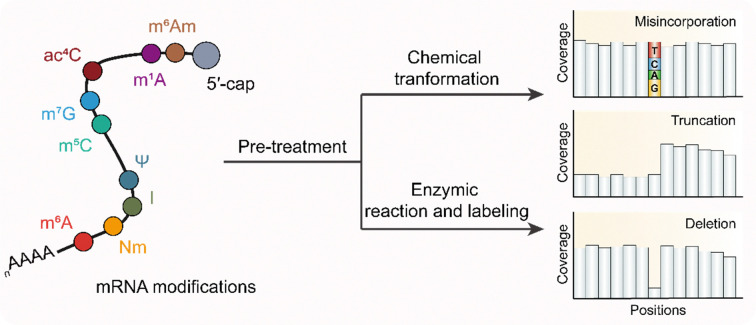

## 1. Introduction

Since their initial discovery in the mid-20th century, many kinds of RNA modifications have been identified, marking a significant boost in the field of epitranscriptomics and enhancing our understanding of transcriptomics. These modifications, found across various RNA species, participate in complex aspects of gene regulation. Originally, the study of RNA modifications mainly focused on abundant non-coding RNAs such as ribosomal RNAs (rRNAs) and transfer RNAs (tRNAs), uncovering their critical functions in RNA biogenesis, stability, translation, and splicing.^1,2^ Recent research studies have extended the landscape to include low abundant and highly divergent RNAs like messenger RNAs (mRNAs) and regulatory non-coding RNAs (ncRNAs), which present challenges for studies using traditional biochemical methods.

Among all RNA modifications, *N*^6^-methyladenosine (m^6^A) stands out as the most abundant internal modification found in mRNAs, alongside other significant modifications like *N*^6^,2′-*O*-dimethyladenine (m^6^Am), *N*^1^-methyladenine (m^1^A), 5-methylcytosine (m^5^C), *N*^4^-acetylcytidine (ac^4^C), *N*^7^-methylguanosine (m^7^G), pseudouridine (Ψ), inosine (I) and 2′-*O*-methylation (Nm).^3–12^ These modifications are known to influence key regulatory processes including transcription, translation, RNA stability and other fate determination processes.^2,7,9,11–29^

The advent of next-generation sequencing (NGS) has enabled the finding of these modifications globally, helping us to understand their biological functions. The goal of ideal sequencing methods is to accurately identify the locations and stoichiometries of RNA modifications. Although antibody-based enrichment followed by sequencing has been a basic and fundamental technique for studying these modifications, it often falls short in terms of accuracy and stoichiometry, which could give us a false sense of their precise location and proportion.^30–33^ Additionally, antibody-based RNA modification sequencing methods often exhibit biases due to non-specific and off-target binding, which can also introduce sequencing bias.^34^ In this review, we summarize the current research progress of these reactivity-based next-generation sequencing methods, addressing the challenges they face and discussing their application in functional studies of the epitranscriptome (Table S1, ESI†).

## 2. mRNA modifications

The central dogma of molecular biology posits that genetic information flows from DNA to RNA, and then to functional proteins. RNA, which exists in various forms such as mRNA, tRNA, rRNA, long non-coding RNA (lncRNA), and small RNA (sRNA), performs diverse roles. Beyond its role in carrying genetic sequences, RNA is subject to many covalent modifications that play critical roles in gene regulation. These modifications introduce an additional layer of RNA function, serving as a dedicated mechanism, with advancements in detection techniques enhancing our exploration of their functional significance.

Each chemical modification has a distinct regulatory impact on RNA metabolism and the overall function. For example, m^6^A, the predominant internal modification in mRNA, modulates various aspects such as transcription, splicing, nuclear export, stability, translation, and even secondary structure.^6,35–49^ On the other hand, m^6^Am is found in the first position adjacent to the 5′ cap structure in many mammalian mRNA molecules. In mRNA, m^1^A distributes mainly in 5′ UTR and regulates translation by altering the RNA structure of translation initiation sites.^9,10^ The internal m^7^G modification enhances mRNA translation efficiency, while ac^4^C in mRNA affects translation.^25–27,50–54^ Within mRNA, m^5^C affects RNA export, stability, and also translational regulation.^15,55^ Moreover, the introduction of Ψ into mRNA not only increases protein production but also modifies translation dynamics.^20,56–58^ The A-to-I editing events influence many layers of gene regulation, such as amino acid alteration, translation, alternative splicing, nuclear retention and nonsense-mediated mRNA decay (NMD).^59–63^ Additionally, Nm may play essential roles in translation and RNA splicing.^64–66^

**Fig. 1:**
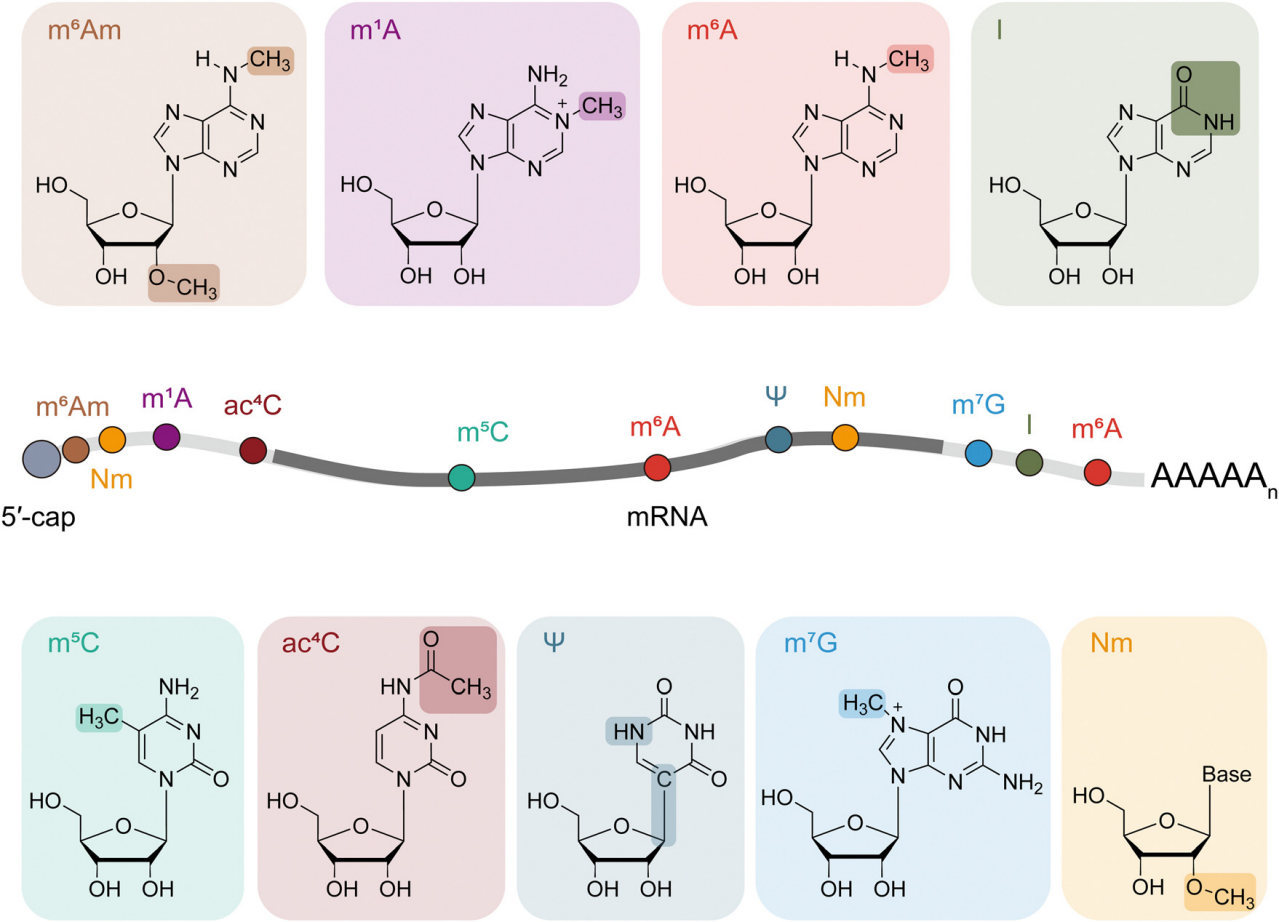
Distribution and chemical structure of mRNA modifications discussed in this review.

The evolution of RNA modification detection technologies has significantly contributed to our comprehensive understanding of the epitranscriptome and facilitated downstream functional studies. With the advent of next-generation sequencing, numerous sequencing technologies have been developed to map modified nucleotides across the transcriptome, unlocking their regulatory functions of RNA modifications. The distinction between modified and unmodified nucleotides enables antibody-based recognition and enrichment for modifications like m^6^A, m^6^Am, m^1^A, m^5^C, ac^4^C and also m^7^G.^9,10,23,25,26,53,67–75^ However, aside from the typically high cost of antibody-related products, challenges also arise with modifications that share similar structures, such as m^6^A and m^6^Am, which cannot be differentiated through antibody enrichment.^67–69^ Even for a single type of RNA modification, antibodies can exhibit non-specific cross-reactivity and batch-to-batch variability. This limitation has driven the development of new strategies for mapping RNA modifications through reactivity-based strategies with specific enzymes and/or chemicals. The following sections include detection methods concentrating on nine important and distinct modifications (m^6^A, m^6^Am, m^1^A, m^5^C, ac^4^C, m^7^G, Ψ, I, and Nm) on mRNAs (Fig. 1 and Table S1, ESI†), which have been highly studied, highlighting the evolution made in this leading field.

## 3. Sequencing methods

### 3.1 m^6^A detection methods

To date, m^6^A methylation sites have been mapped to the transcriptome, predominantly located in long exons, near stop codons, and mostly in 3′ untranslated regions (3′ UTRs) in mammalian cells.^67,68^ In this section, we will primarily summarize those reactivity-based methods for locus-specific m^6^A detection and quantification.

#### 3.1.1 Brief introduction of antibody-based m^6^A sequencing methods

Currently, various m^6^A sequencing detection methods have been developed. The earliest method is based on m^6^A-specific antibody immunoprecipitation sequencing. This method was first developed simultaneously in two independent studies and was named methylated RNA immunoprecipitation sequencing (MeRIP-seq, or m^6^A-seq).^67,68^ The principles and methods of both are essentially the same: mRNA is fragmented and then incubated with m^6^A antibodies to achieve immunoprecipitation enrichment of m^6^A-containing fragments. Subsequent methods for m^6^A detection have effectively improved the resolution of m^6^A-seq, allowing for the detection of m^6^A positions with even single-nucleotide resolution. In PA-m^6^A-seq, 4-thiouridine (4sU) is a photoactivatable nucleotide analog that can be metabolized into newly synthesized RNA by mimicking uridine (U).^76^ Under UV light excitation, the carbon–sulfur double bond undergoes free dissociation and reacts with other molecules such as amino acids in proteins. It improves the resolution of m^6^A peaks (up to 23 nucleotides) by covalently crosslinking 4sU-labeled RNA and m^6^A antibodies. Moreover, to address the challenge of mapping m^6^A modifications at single-nucleotide resolution, researchers have developed miCLIP methods.^77^ These methods use 254 nm UV light instead of 4sU metabolic labeling. These techniques crosslink m^6^A-containing RNA with anti-m^6^A antibodies and induce mutations or truncations during reverse transcription (RT) to achieve single-nucleotide identification of m^6^A modification sites. These antibody-based m^6^A detection techniques have significantly advanced the study of m^6^A-related functions. However, issues such as antibody specificity and low immunoprecipitation efficiency, which require large sample inputs, still need to be addressed. They also face challenges such as low efficiency and difficulty in maintaining consistent enrichment levels across multiple experiments. Developing rapid and accurate high-throughput sequencing methods remains a challenge.

#### 3.1.2 Enzyme-assisted m^6^A sequencing methods

**Fig. 2:**
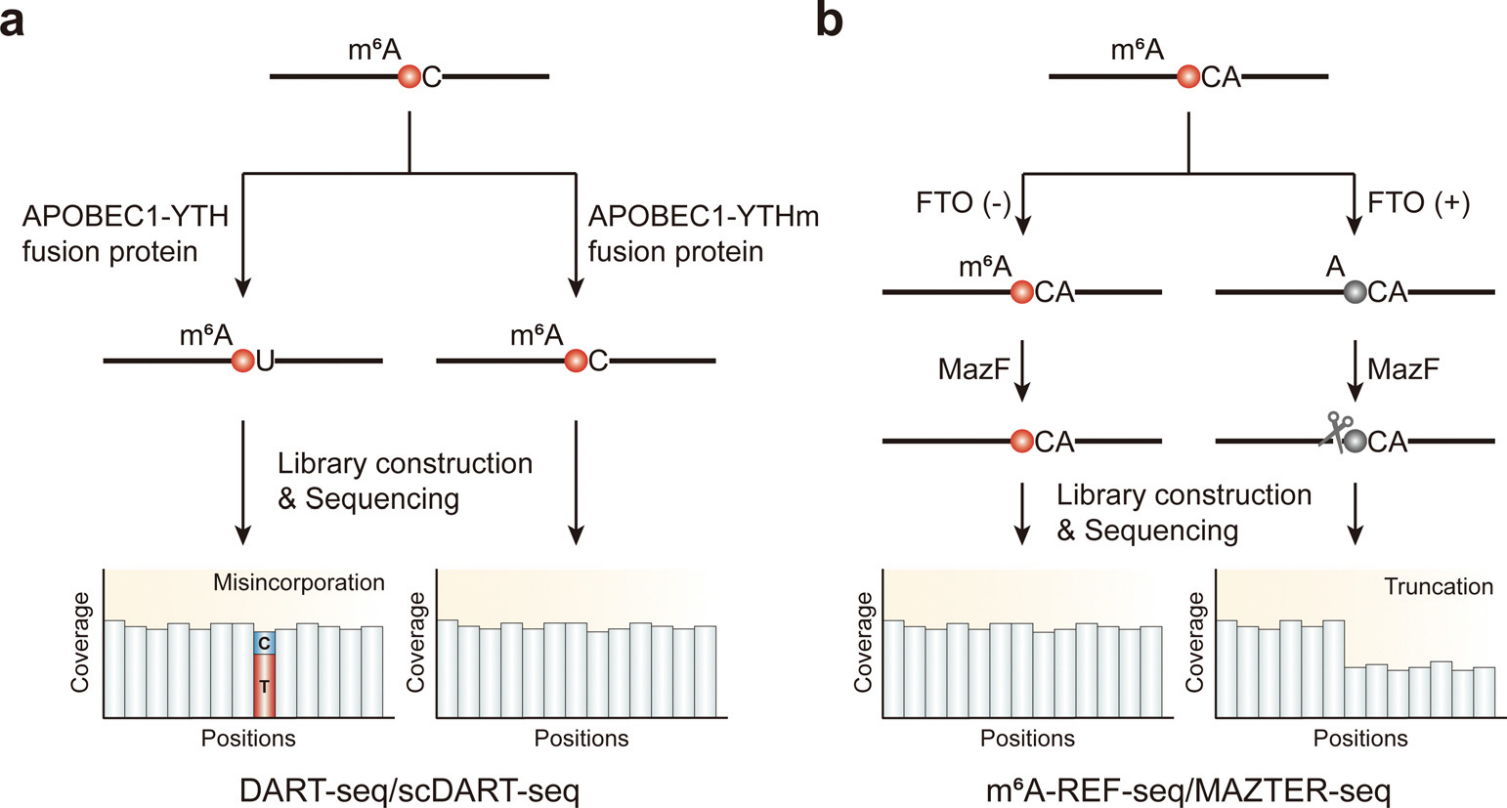
Overview of enzyme-assisted sequencing methods for the detection of m^6^A modifications. (a) DART-seq and scDART-seq. (b) m^6^A-REF-seq and MAZTER-seq.

The YT521-B homology (YTH) domain has been identified as a specific recognizer of the m^6^A modification, enabling YTH-domain-containing proteins, such as YTHDF1-3 and YTHDC1-2 in mammals, to engage in various biological functions through m^6^A recognition.^39,41,78–82^ Resorting to this specific interaction, DART-seq emerges as an innovative, antibody-free technique for comprehensive m^6^A detection.^83^ In DART-seq, the YTH domain is fused with cytidine (C) deaminase APOBEC1^83^ connecting with a short linker. This APOBEC1-YTH fusion protein induces C to U deamination at sites adjacent to m^6^A residues, with these mutations subsequently detected through RNA sequencing. To identify the specificity of these mutations to m^6^A sites, a mutant APOBEC1-YTH variant (APOBEC1-YTHm) lacking m^6^A-binding capability serves as a negative control, helping to reduce false positive signals (Fig. 2a). DART-seq, which can directly sequence mRNAs without immunoprecipitation enrichment, needs only a low RNA input, exhibiting great promise for detecting m^6^A at the single-cell level. In recent progress, DART-seq was combined with a single-cell RNA-sequencing platform to create scDART-seq, allowing for the profiling of the m^6^A methylome at the single-cell level.^84,85^

DART-seq has the unique function of irreversibly marking m^6^A sites over several hours allowing APOBEC1-YTH to access and edit structurally hidden sites under physiological conditions. Therefore, it identifies a broader range of sites than antibody-based methods. Moreover, DART-seq has the ability to monitor different mutation sites within individual transcripts, allowing for the determination of m^6^A sites’ presence within the same transcript by long-read sequencing.^83^ Potential applications of DART-seq include m^6^A profiling in various cell types under different physiological states and the detection of m^6^A within specific cellular compartments by incorporating localization elements into the APOBEC1-YTH fusion. However, it relies on the *in vivo* overexpression of APOBEC1-YTH, which limits its efficiency for *in vitro* applications in transfection-challenging materials. Additionally, with only 60% RNA substrates targeted by the YTH domain containing m^6^A, false negatives present a challenge that may be addressed by enhancing the affinity and specificity of the YTH domain. Besides that, C-to-U base editing can result in unfavorable gene expression, potentially leading to protein dysfunction, which could influence cellular homeostasis.

MazF, identified as a methylation-sensitive endoribonuclease, uniquely cleaves the unmethylated ACA motif but not the methylated (m^6^A)CA motif. Using this specificity, two similar methods, m^6^A-REF-seq and MAZTER-seq,^86,87^ have been developed. These techniques involve treating parallel samples—comparing either control cells to m^6^A writer knockout cells, where METTL3, as a crucial methyltransferase, is essential for m^6^A modification, or RNA subjected to FTO demethylation reactions *versus* untreated RNA—with MazF, followed by RNA sequencing. Ideally, after MazF treatment, RNA fragments should initiate at an ACA site and end just before the next ACA site, allowing reads to span m^6^A sites (Fig. 2b). The presence of m^6^A inversely correlates with cleavage efficiency, enabling the identification and quantification of m^6^A sites using the MAZTER-MINE computational pipeline.^86^ This pipeline calculates cleavage efficiencies at the 5′ and 3′ ends from RNA-seq data to estimate m^6^A abundance at specific sites. By analyzing ACA sequences in paired samples, these methods can pinpoint transcriptomic m^6^A sites within these specific motifs and quantify methylation levels with single-base precision.

The high sensitivity and specificity of MazF, combined with a straightforward experimental procedure without antibody enrichment, make these methods particularly suited for limited samples, including those from pathological tissues or early embryos. However, due to the exclusive recognition of the ACA motif, only partial m^6^A sites can be detected. Factors such as the secondary structure of RNA, MazF enzyme activity, and sequence preference may influence result accuracy; on the other hand, the quantitation also needs to be challenged by the limited enzymatic efficiency of MazF.^34^ Nevertheless, the potential to uncover more sites exists, either by finding additional enzymes with different motif specificities or by optimizing current enzymes to recognize a broader set of m^6^A methylation sites.

#### 3.1.3 Combined enzyme- and chemical-assisted m^6^A sequencing methods

**Fig. 3:**
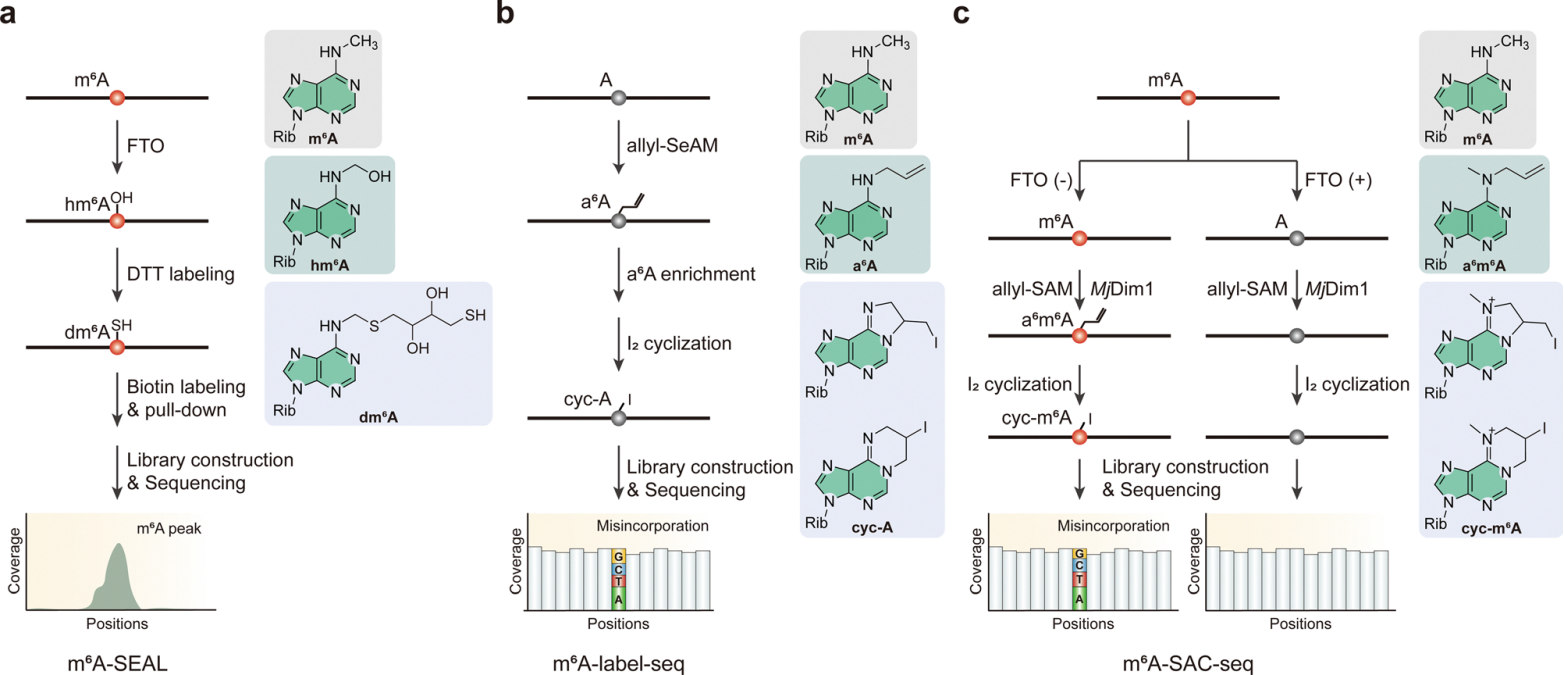
Overview of enzyme- and chemical-assisted sequencing methods for the detection of m^6^A modifications. (a) m^6^A-SEAL. (b) m^6^A-label-seq. (c) m^6^A-SAC-seq. Rib, ribose. DTT, dithiothreitol. Allyl-SeAM, *Se*-allyl-adenosyl methionine. Allyl-SAM, *S*-allyl-adenosyl methionine.

Researchers have also devised several m^6^A sequencing methods that make use of enzymic reactions combined with further chemical labeling. In 2020, m^6^A-SEAL represented a significant improvement in the specific detection of m^6^A across the transcriptome (Fig. 3a).^88^ This method takes advantage of FTO to oxidize m^6^A to *N*^6^-hydroxymethyladenosine (hm^6^A) and subsequently to *N*^6^-formyladenosine (f^6^A) under physiologically conditions.^89^ Moreover, this process resorts to the relatively rapid conversion of m^6^A to hm^6^A and the slower rate to f^6^A, with hm^6^A reacting with dithiothreitol (DTT) to form a stable *N*^6^-dithiothreitolmethyladenosine (dm^6^A) (Fig. 3a). This specific chemical reaction introduces an exposed sulfhydryl group (–SH) that can be tagged by biotin derivative and further enriched *via* streptavidin immunoprecipitation, allowing for the identification of transcripts containing m^6^A by comparison with an untreated input sample.

Due to the high specificity of FTO to oxidize the methyl group of m^6^A, m^6^A-SEAL minimizes false-positive signals compared to MeRIP-seq, offering an antibody-free method with high sensitivity, specificity, and reliability for mapping m^6^A transcriptome-wide. Interestingly, both m^6^Am and m^6^A serve as FTO substrates, but FTO exhibits greater reactivity with m^6^Am *in vitro*. This differential reactivity means the potential to distinguish between m^6^A and m^6^Am by fine-tuning the oxidation condition. Moreover, given that FTO can also oxidize DNA 6mA to *N*^6^-hydroxymethyldeoxyadenosine (d6mA) under certain conditions, it has the potential for adapting m^6^A-SEAL for DNA 6mA detection. While m^6^A-SEAL has a base resolution comparable to MeRIP-seq (about 200 nt), achieving single-base resolution could be possible by optimizing the reverse transcription process to induce truncations or mutations near the dm^6^A site in order to enhance the accuracy in mapping m^6^A modifications under the single-base resolution.^67,68^

In addition to *in vitro* reaction and labeling strategies, researchers have also developed metabolic labeling methods for single-base m^6^A detection. The biogenesis of m^6^A in mRNA involves the transfer of a methyl group from *S*-adenosyl methionine (SAM) to specific adenosine (A) sites within RNA by the m^6^A methyltransferase complex, a central process similar to many fundamental biological processes.^90^ However, the inherent chemical stability of the methyl group on m^6^A and its consistent base pairing pattern with A present substantial challenges for precise detection by high-throughput sequencing techniques, perplexing the accurate mapping of m^6^A modifications throughout the transcriptome. To address these challenges, researchers have developed a metabolic labeling approach known as m^6^A-label-seq (Fig. 3b).^91^ This method enables transcriptome-wide, single-base resolution detection of m^6^A by substituting the methyl group with an allyl group. This substitution is facilitated by feeding cells with *Se*-adenosyl-l-selenomethionine, a small-in-size methionine analog, which leads to the metabolic incorporation of an allyl group into specific adenosine sites, producing a modified nucleotide termed *N*^6^-allyladenosine (a^6^A).^92–94^ Due to the structural similarity of the isopentenyl and allyl groups, labeled a^6^A allows for selective enrichment by commercial *N*^6^-isopentenyladenosine (i^6^A) antibodies. The a^6^A modification then undergoes specific iodination (I_2_)-induced cyclization to form *N*^1^,*N*^6^-cyclized adenosine (cyc-A), leading to misincorporations during reverse transcription because of steric hindrance, thereby enabling precise identification of m^6^A sites.

Despite its precision, m^6^A-label-seq identifies fewer sites compared to other methods, attributed to low incorporation efficiency and the associated loss of quantitative information. This highlights the necessity for methodological advancements to enhance both incorporation and chemical transformation efficiencies. Future improvements might involve engineering more efficient methionine adenosyl methyltransferases for increased a^6^A yield or refining reverse transcriptase enzymes to boost mutation efficiency during sequencing. Additionally, the application of *Se*-adenosyl-l-selenomethionine could induce cellular stress, potentially influencing sequencing results from the bottom up. Thus, further refinement of the method is required to reduce such side effects and improve result reliability.

Similar to m^6^A-label-seq, m^6^A-SAC-seq provides a selective and quantitative strategy for high-resolution mapping of m^6^A across the transcriptome (Fig. 3c).^95^ Both techniques utilize the chemical reactivity of the allyl group, with m^6^A-SAC-seq uniquely comprising an *in vitro* enzymatic reaction by *Mj*Dim1 (a homolog of Dim1 in *M. jannaschii*) for allyl labeling at m^6^A sites.^91,95^ This process involves the enzymatic transfer of an allyl group from allyl-SAM to both m^6^A and A, resulting in the formation of a^6^m^6^A and a^6^A, respectively. Subsequent iodination-induced cyclization causes base misincorporation during reverse transcription, thereby enabling precise m^6^A identification at the single-nucleotide level without the need for enrichment.

The selectivity of *Mj*Dim1-catalyzed allyl transfer from allylic-SAM is approximately tenfold higher for m^6^A than A, with the human immunodeficiency virus (HIV) reverse transcriptase inducing higher mutation rates at the labeled and cyclized a^6^m^6^A sites compared to cyclized a^6^A sites. One of the key advantages is its minimal RNA input requirement, making it highly adaptable for studies with limited sample availability. Additionally, using RNA samples treated with the m^6^A demethylase FTO as a background control enhances the specificity of this method, allowing for the differentiation of true m^6^A modifications from background noise. Another significant benefit of m^6^A-SAC-seq is its capability for m^6^A quantification through mutation rate correlation. This feature makes it possible to acquire quantitative data on m^6^A levels, offering insights into the dynamic modification proportion under various conditions. However, the method does exhibit certain limitations, including a motif preference for GAC over AAC, which may result in the under-detection of some m^6^A sites, especially for the m^6^A quantitation. Although it can identify approximately 80% of m^6^A sites—largely due to the prevalence of the GAC motif among 70–75% of m^6^A sites—it faces challenges in detecting AAC sites. Moreover, the requirement for higher sequencing depth compared to antibody-based methods may restrict its widespread use in settings with limited sequencing capabilities.

#### 3.1.4 Detection of m^6^A sites using nitration and/or deamination reaction

**Fig. 4:**
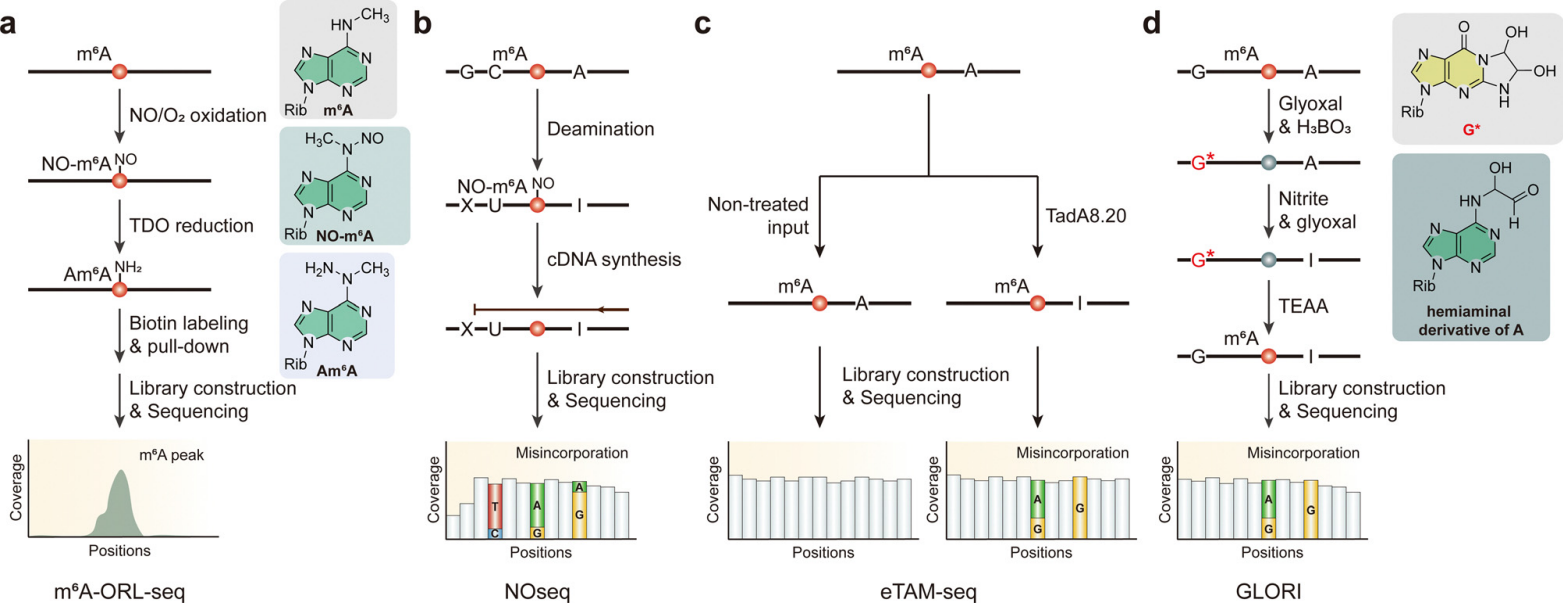
Overview of sequencing methods for the detection of m^6^A modifications using nitration and/or deamination reaction. (a) m^6^A-ORL-seq. (b) NOseq. (c) eTAM-seq. (d) GLORI. Rib, ribose. TDO, thiourea dioxide. TEAA, triethylamine acetate.

A recent study has introduced m^6^A-ORL-seq, a novel chemical method for detecting m^6^A in RNA with satisfactory resolution and specificity (Fig. 4a).^96^ m^6^A-ORL-seq employs a three-step chemical reaction—oxidation, reduction, and functional labeling—to identify m^6^A sites at single-base resolution. The experimental approach validates the method using various RNA oligos and HEK-293T cell RNA, demonstrating high specificity and efficiency in m^6^A detection. This method successfully identifies 4000 high-confidence m^6^A sites in the human transcriptome. It can detect low-level m^6^A modifications often missed by other techniques, highlighting its sensitivity. The antibody/enzyme-free nature makes it a cost-effective and scalable option for transcriptome-wide m^6^A profiling. This new technique can be applied to study m^6^A modifications across different RNA types and biological conditions, offering a valuable tool for exploring the roles of RNA modifications in cellular processes. Future improvements could further enhance its efficiency and mutation rates, potentially enabling quantitative analysis of m^6^A levels.

Similar to m^6^A-ORL-seq, NOseq is a method for detecting m^6^A by exploiting its resistance to chemical deamination by nitrous acid (Fig. 4b).^97^ This innovative technique includes a deamination process, which converts cytidine to uridine, adenosine to inosine (I), guanine (G) to xanthosine (X), and m^6^A to *N*^6^-methyl-*N*^6^-nitrosoadenosine (NO-m^6^A) while leaving uridine unaltered. The resulting sequence changes are analyzed using a specialized mapping algorithm designed to handle the sequence degeneration caused by deamination, ensuring precise detection of m^6^A sites. NOseq was experimentally validated by detecting known m^6^A sites in human rRNA and lncRNA MALAT1, as well as several candidate m^6^A sites in the *Drosophila melanogaster* transcriptome. The method proved effective in identifying m^6^A with partial modification levels around 50%, and this threshold could be lowered to approximately 10% when combined with m^6^A immunoprecipitation. Although NOseq represents a significant advancement in m^6^A detection, offering us a powerful tool for exploring RNA modifications and their biological functions, future improvements could further enhance the reaction efficiency and reduce the RNA degradation affected by nitrous acid.

Evolved TadA-assisted *N*^6^-methyladenosine sequencing (eTAM-seq) is a technique designed to achieve high-resolution profiling and quantification of m^6^A across the transcriptome (Fig. 4c). This method uses enzyme-assisted adenosine deamination to detect and quantify m^6^A modifications with exceptional precision.^98^ Central to eTAM-seq is the use of a hyperactive variant of the TadA enzyme, TadA8.20, which selectively converts unmethylated A to I, while leaving m^6^A sites unaltered. During reverse transcription, inosines are recognized as G, allowing for the identification of m^6^A as persistent adenosine signals. This approach facilitates not only transcriptome-wide m^6^A mapping but also site-specific quantification with minimal RNA input, making it a powerful tool for epitranscriptomic studies.

One of the primary advantages of eTAM-seq is its ability to provide base-resolution mapping of m^6^A sites. This high level of precision enables the accurate localization of m^6^A modifications across the transcriptome, which is critical for understanding the functional roles of these modifications in gene expression regulation. Additionally, eTAM-seq is characterized by its low input requirement, capable of detecting and quantifying m^6^A modifications with as few as ten cells or 250 picograms (pg) of total RNA. This sensitivity represents a significant improvement over traditional methods, which often necessitate much larger quantities of RNA. Another key benefit of eTAM-seq is its preservation of RNA integrity. Unlike chemical deamination methods, which can degrade RNA and compromise the accuracy of results, eTAM-seq employs an enzymatic approach that maintains the structural integrity of RNA, reducing the risk of sample loss and ensuring more reliable data.^96,97^ Furthermore, eTAM-seq offers quantitative capabilities, allowing researchers to not only detect the presence of m^6^A modifications but also quantify the extent of methylation at specific sites. eTAM-seq can be adapted for various applications, including potential single-cell m^6^A profiling, which could provide unprecedented insights into the heterogeneity of m^6^A modifications at the individual cell level.

Despite its many advantages, eTAM-seq has certain limitations. The efficiency of the deamination process is partially dependent on the RNA secondary structure. Highly structured RNA regions may obstruct the accession of enzyme, leading to incomplete deamination and potentially resulting in false negatives. Additionally, the method may yield false-positive signals due to other adenine modifications that resist deamination by the enzyme, although this issue can be mitigated by using demethylases like FTO to confirm confident m^6^A sites. Another limitation of eTAM-seq is its reduced sensitivity to low methylation levels. This method may not accurately detect m^6^A sites with methylation levels below 25%, which could result in the underrepresentation of certain modifications in the data. Moreover, the accuracy of m^6^A mapping using eTAM-seq requires the use of control samples to estimate site accessibility, adding an extra layer of complexity to the experimental workflow.

Given that the reaction efficiency of m^6^A-SAC-seq varies depending on the motif surrounding each m^6^A site, the lack of quantitative information from m^6^A-ORL-seq/NOseq, and the potential for false negatives and inefficiency in detecting low methylation level sites using deamination enzyme, significant progress needs to be made in the pursuit of more accurate m^6^A detection. Existing methods often face challenges such as limited site-specific resolution, motif biases, and the complexities associated with sophisticated experiment and computational analysis.^83,86–88,95^ However, GLORI represents a breakthrough in m^6^A detection technology, overcoming these hurdles to achieve precise, single-base identification and quantification of m^6^A sites (Fig. 4d).^99^ Using a catalytic system discovered through screening combinations of chemical reactions, nitrite can efficiently deaminate unmethylated adenosine into inosine, achieving an A-to-I conversion rate surpassing 98%. In this system, glyoxal reacts with guanosine in borate buffer to protect the exocyclic amino group forming a glyoxal-guanosine adduct (G*) and with adenosine at the *N*^6^ position to generate a *N*-(hydroxymethylene) hemiaminal derivative, which acts as a catalyst in the deamination process. During reverse transcription, inosine pairs with cytidine and is subsequently read as guanosine in sequencing, resulting in an A-to-G conversion. In contrast, m^6^A is unaffected and remains identifiable as adenosine. This method allows for the absolute quantification of m^6^A at the single-base level by assessing the proportion of A in sequencing reads, allowing this chemical reaction to distinguish between methylated and unmethylated adenosines accurately.

GLORI has a strong ability to detect m^6^A accurately, which makes it a valuable tool in m^6^A functional research. GLORI sets itself apart with its antibody-free, highly sensitive approach, capable of detecting even low levels of m^6^A modifications with high technical repeatability. It consistently identifies the canonical DRAC motif (D = G/A/T, R = A/G) in m^6^A sites and has been applied in studies of dynamic m^6^A regulation under stress conditions. These findings demonstrate GLORI has the ability to explore the function of m^6^A in essential biological processes and stress responses. However, the transformation efficiency of the chemical reaction between nitrite and adenosine is largely influenced by the length of the transcripts. Moreover, despite its high A-to-I conversion rate, the treatment with glyoxal and nitrite leads to the degradation of RNA into relatively short chains of nucleic acids, complicating sequencing and data analysis.

### 3.2 m^6^Am detection methods

m^6^Am is a 5′-terminal modification found at the first nucleotide following the mRNA cap. When adenine is the first transcribed nucleotide in mammalian mRNAs, it can undergo a co-transcriptional methylation to form *N*^6^,2′-*O*-dimethyladenosine.^77,100,101^ The relative content of this modification is approximately one-tenth of that of m^6^A. Given its specific location at the first base of mRNA, m^6^Am has a potential role in regulating translation. Notably, this modification is not exclusive to mRNAs; m^6^Am is also found internally within U2 snRNAs.^102^

High-throughput sequencing methods, combined with the immunoprecipitation of fragmented RNAs using m^6^A-specific antibodies, have been developed to identify m^6^Am-containing RNAs.^67–69^ However, these methods have a significant limitation—anti-m^6^A antibodies cannot distinguish between m^6^Am and m^6^A. This makes sequencing techniques like m^6^A-seq/MeRIP-seq and methylation iCLIP (miCLIP) less effective for accurate m^6^Am mapping. To directly identify m^6^Am, researchers have developed CAPturAM, a novel antibody-free chemical biology approach that directly enriches and probes physiological PCIF1 targets. In this method, cap-m^6^Am is enzymatically propargylated using PCIF1 with a synthetic AdoMet analog. The propargylated m^6^Am is then selectively biotinylated and enriched using magnetic streptavidin beads. This strategy is expected to significantly enhance transcriptome-wide studies by identifying PCIF1 targets and m^6^Am sites. Despite its promising potential, CAPturAM currently faces certain limitations, including residual internal background propargylation, even after stringent optimization of enzymatic modification conditions. It is crucial to address this issue to improve the specificity and accuracy. Future advancements in CAPturAM are expected to incorporate RNA-seq, which would present an antibody-free technique for the direct and comprehensive identification of m^6^Am sites across the transcriptome.

### 3.3 m^1^A detection methods

The *N*^1^ site of the adenine base undergoes methylation to form the m^1^A modification. Intriguingly, m^1^A possesses a positive charge under physiological conditions. The presence of the *N*^1^ methyl group modifies the structure of the base, influencing its free energy, the manner it pairs with other bases, and the mechanics of pairing. Notably, m^1^A can engage in Hoogsteen base pairing, potentially leading to mismatches during the reverse transcription process.^103^ Comprehensive investigation of m^1^A heavily relies on the development of detection methodologies, which have progressed from traditional chemical analysis to modern high-throughput sequencing techniques.

#### 3.3.1 Detection of m^1^A methylation with demethylation

**Fig. 5:**
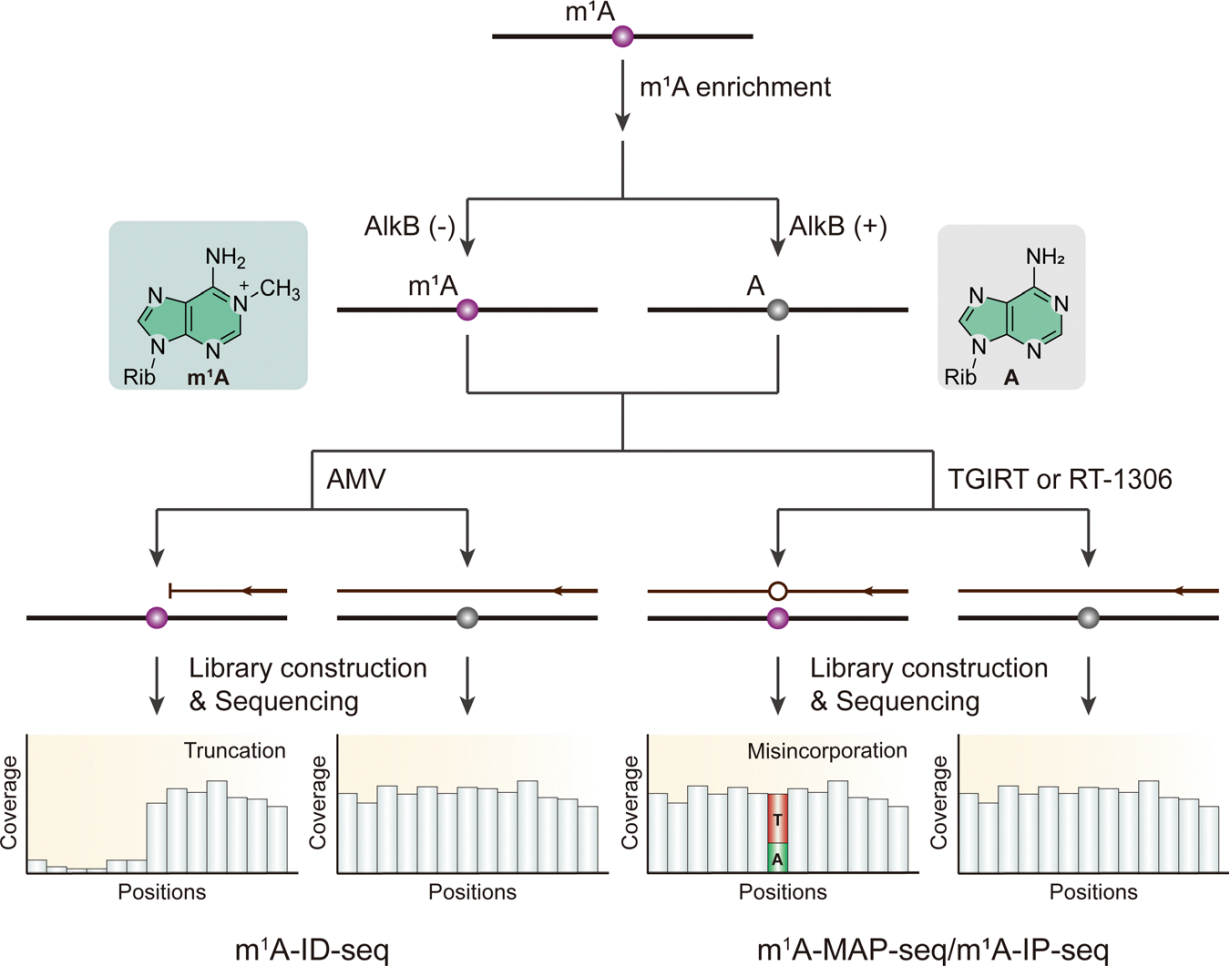
Overview of sequencing methods for the detection of m^1^A modifications using demethylase AlkB. Rib, ribose. AMV, avian myeloblastosis virus reverse transcriptase. TGIRT, thermostable group II intron reverse transcriptase.

In response to these challenges, researchers developed m^1^A-ID-seq (Fig. 5), which integrates m^1^A immunoprecipitation with m^1^A-induced truncated reverse transcription products using avian myeloblastosis virus (AMV) reverse transcriptase.^104^ This technique enabled the first comprehensive, transcriptome-wide characterization of m^1^A, revealing a reversible and dynamic m^1^A methylation program in the human transcriptome. This method provides valuable tools for exploring the functional roles of m^1^A in biological regulation.

However, the truncated complementary DNA (cDNA) synthesis might lead to the loss of information of the m^1^A methylation. Like m^1^A-ID-seq, m^1^A-MAP-seq and m^1^A-IP-seq combines enzymatic demethylation and reverse transcription under different conditions using thermostable group II intron reverse transcriptase (TGIRT) and RT-1306, respectively (Fig. 5).^23,70^ RT-1306, an engineered novel reverse transcriptase, yields a tenfold increase in full-length cDNA production and a higher ratio of reads to truncated products compared to TGIRT.^70^ Demethylated RNA is subjected to RT to generate cDNA, followed by library preparation for subsequent comparison. Untreated RNA, on the other hand, induces misincorporation by TGIRT or RT-1306. Therefore, the precise location of the m^1^A modification can be determined, facilitating the identification of m^1^A modification sites with single-nucleotide resolution. m^1^A-quant-seq, modified from m^1^A-IP-seq, incorporates synthetic m^1^A oligonucleotides for estimating m^1^A stoichiometry.^70^ However, reliance on demethylation treatment can lead to false negatives, particularly when RNA methylation abundance is low or when the methylation site is located within complex structures that resist demethylation processes. Moreover, the calibration curve conforms to a nonlinear equation, indicating that RT-1306 may still cause some degree of truncation in biological RNA samples. This truncation reduces sensitivity at certain sites, complicating the accurate detection and mapping of modifications.

#### 3.3.2 Detection of m^1^A methylation with Dimroth rearrangement

**Fig. 6:**
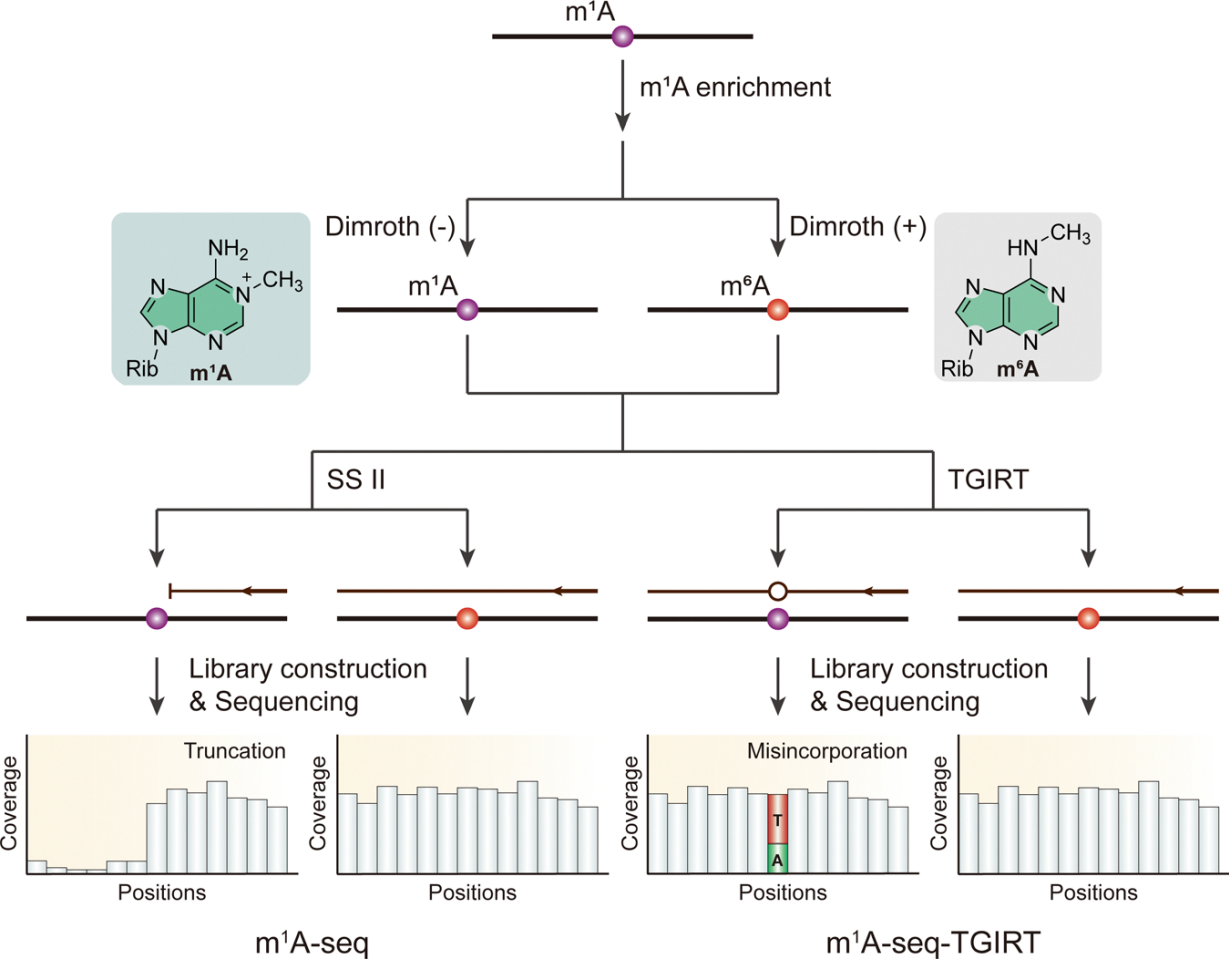
Overview of sequencing methods for the detection of m^1^A modifications using Dimroth rearrangement. Rib, ribose. SS II, SuperScript II. TGIRT, thermostable group II intron reverse transcriptase.

By looking at the distinct chemical feature of m^1^A, considering that m^1^A can be converted into m^6^A under alkaline conditions through a process known as Dimroth rearrangement (DR), researchers have developed m^1^A-seq, a method that involves treating precipitated m^1^A-containing mRNA fragments with an alkaline buffer to chemically rearrange m^1^A to m^6^A before cDNA synthesis (Fig. 6).^71^ By comparing mismatch rates between treated and untreated samples, researchers can locate m^1^A positions within m^1^A peaks, achieving a resolution of 5–15 nucleotides. In some cases, conserved m^1^A sites in rRNA can be mapped at single-nucleotide resolution. Furthermore, research has revealed that, compared to the m^1^A-seq alone, the combined utilization of TGIRT results in a higher incorporation rate and lower truncation rate, whereas the use of SuperScript II (SS II) reverse transcriptase yields a higher truncation rate but a lower false incorporation rate (Fig. 6).^71^ Both methods exhibit enhanced sensitivity and specificity in detecting m^1^A modifications, leading to a reduction in false-positive rates and a more accurate estimation of m^1^A stoichiometry. However, both strategies, which cause high RNA degradation, sacrifice sequencing signal and may lose some sequence information near the modified sites. This limitation poses a significant challenge in obtaining high-quality, single-base resolution maps of m^1^A. Recently, researchers have developed a mild chemical catalysis method using 4-nitrothiophenol under slightly acidic conditions. This approach results in both low degradation ratio and higher rearrangement efficiency compared to the traditional Dimroth reaction.^105^ Thus, combining this chemical reaction and these m^1^A sequencing methods above could achieve a better level of m^1^A detection.

### 3.4 m^5^C detection methods

Although methylated cytosine residues at position 5 (5mC) are very common in DNA, m^5^C in RNA did not initially gain much attention due to its lower abundance. Previous methods for detecting m^5^C in RNA, such as high-performance liquid chromatography (HPLC) and mass spectrometry (MS), required exceedingly high amounts of RNA and could only reliably identify methylated sites in highly abundant and stable ncRNAs, such as tRNAs and rRNAs.^106,107^ However, with the development of high-throughput sequencing approaches, m^5^C has been found to be widely distributed in mRNA.^108^ Notably, m^5^C is often located near argonaute-binding regions within the 3′ UTR or in the vicinity of the translational start site of mRNA.^8,16^

**Fig. 7:**
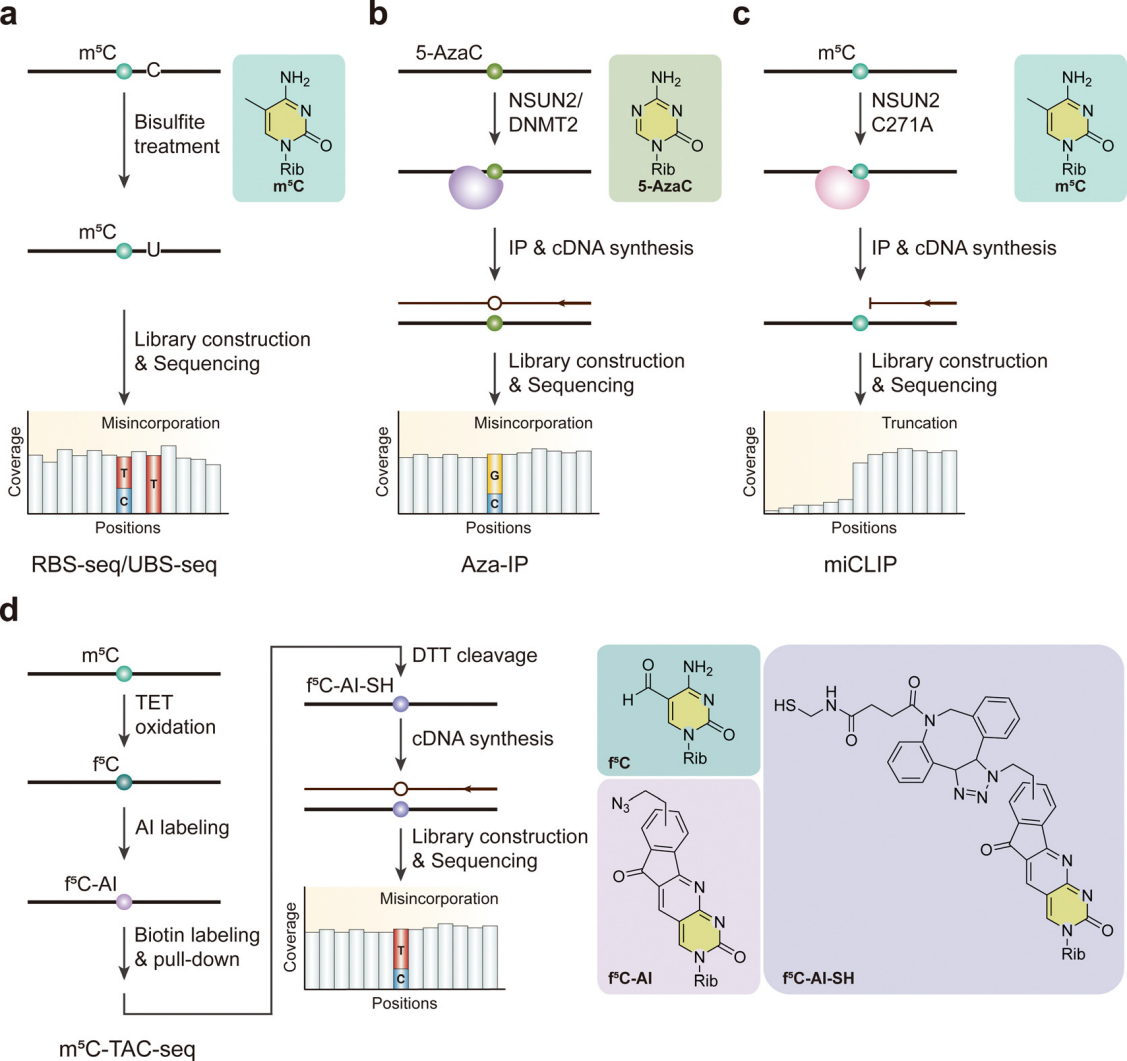
Overview of sequencing methods for the detection of m^5^C modifications. (a) RBS-seq. (b) Aza-IP. (c) miCLIP. (d) m^5^C-TAC-seq. Rib, ribose. TET, ten-eleven translocation methylcytosine dioxygenase. AI, 1,3-indandione. DTT, dithiothreitol.

Bisulfite sequencing (BS-seq), a gold standard method for detecting m^5^C in genomic DNA (gDNA), is based on the chemical deamination of cytosines with sodium bisulfite (NaHSO_3_) treatment and has been applied to mRNA.^109,110^ Sodium bisulfite deaminates unmethylated cytosines into uridines in single-stranded DNA or RNA, while methylated cytosines remain unconverted.^110,111^ During subsequent analysis, unmethylated C is read as T, while methylated C is still read as C. Using bisulfite sequencing, thousands of m^5^C sites in mRNA have been identified in humans.^16^ In general, while bisulfite sequencing can achieve single-base-resolution detection of m^5^C, its limitation in converting cytosines of single-stranded nucleic acids can lead to incomplete conversion in RNA secondary structure regions, resulting in a large number of false-positive m^5^C sites. To address this, RBS-seq, which uses heating and formamide to denature RNA and improves the C-to-U conversion efficiency in double-stranded regions, has been developed (Fig. 7a).^110^ This method has identified 486 candidate m^5^C sites in mammalian mRNA.^112^ Additionally, achieving a high conversion rate requires prolonged incubation under consecutive acidic and alkaline conditions, which also causes RNA degradation. This degradation can compromise the subsequent reverse transcription and PCR amplification steps. To overcome this issue, an ultrafast bisulfite sequencing method (UBS-seq) has been developed for mapping 5-methylcytosine in both DNA and RNA.^113^ UBS-seq optimizes the reaction by using ammonium salts of bisulfite and sulfite and performing the reaction at 98 °C for approximately 10 minutes, affording a substantially lower background than previous approaches.

Two members of the NSUN family, NSUN2 and NSUN6, are responsible for mRNA methylation, with 90% of m^5^C sites being sensitive to NSUN2 depletion and a small fraction being NSUN6 substrates.^113^ Considering NSUN2 methylates the majority of m^5^C sites, two methods have been developed based on the catalytic methylation mechanism. In the 5-azacytidine-mediated RNA immunoprecipitation method (Aza-IP), 5-azacytidine (5-AzaC), a cytidine analog with a nitrogen substitution at carbon 5, is randomly incorporated into nascent RNA by RNA polymerases in cells overexpressing an epitope-tagged m^5^C RNA methyltransferase (Fig. 7b).^114^ The incorporation of 5-AzaC affects the release of methyl transferase at carbon 5, forming a stable covalent bond. After immunoprecipitation, specific C-to-G conversion can be observed at targeted C residues, enabling the detection of m^5^C at single-base resolution. Using Aza-IP, the direct targets of NSUN2 and DNMT2, a tRNA m^5^C methyltransferase, can be identified, revealing specific methylated cytosines. miCLIP has been developed to successfully identify transcriptome-wide m^5^C sites methylated by NSUN2.^73^ This method utilizes the catalytic principle that a cysteine-to-alanine mutation (C271A) in the NSUN2 protein impedes the release of enzymes from the protein–RNA complex. This results in the formation of a stable covalent bond between NSUN2 and its RNA target, causing truncation during reverse transcription and thereby generating single-nucleotide-resolution information (Fig. 7c). While both Aza-IP and miCLIP methodologies are dependent on the formation of covalent bonds between the RNA methylase and its substrate, the accuracy of m^5^C detection methods is still compromised by challenges such as nonspecific antibody binding and mislocalization of methyltransferases. To address this challenge, a BS-free, base-resolution m^5^C detection strategy was enabled by TET-assisted chemical labeling (m^5^C-TAC) (Fig. 7d).^115^ In m^5^C-TAC-seq, m^5^C is first oxidized to f^5^C, then labeled with an azido derivative of 1,3-indandione (AI). This labeling facilitates the enrichment of m^5^C-containing RNAs *via* biotin pull-down and induces C-to-T transitions at m^5^C sites. Importantly, this method is gentle on RNA and does not affect unmodified Cs, enabling the direct detection of m^5^C even in RNAs with low abundance or low sequence complexity.

### 3.5 ac^4^C detection methods

ac^4^C was initially discovered during the characterization of eukaryotic rRNAs and tRNAs.^116,117^ The production of ac^4^C in eukaryotic RNA is unique, relying exclusively on the enzyme *N*-acetyltransferase 10 (NAT10).^28,117^ Through an adenosine triphosphate (ATP)-dependent process, NAT10 executes this function by shifting an acetyl group from acetyl-CoA to the exocyclic *N*^4^-amine of cytidine.^118^

**Fig. 8:**
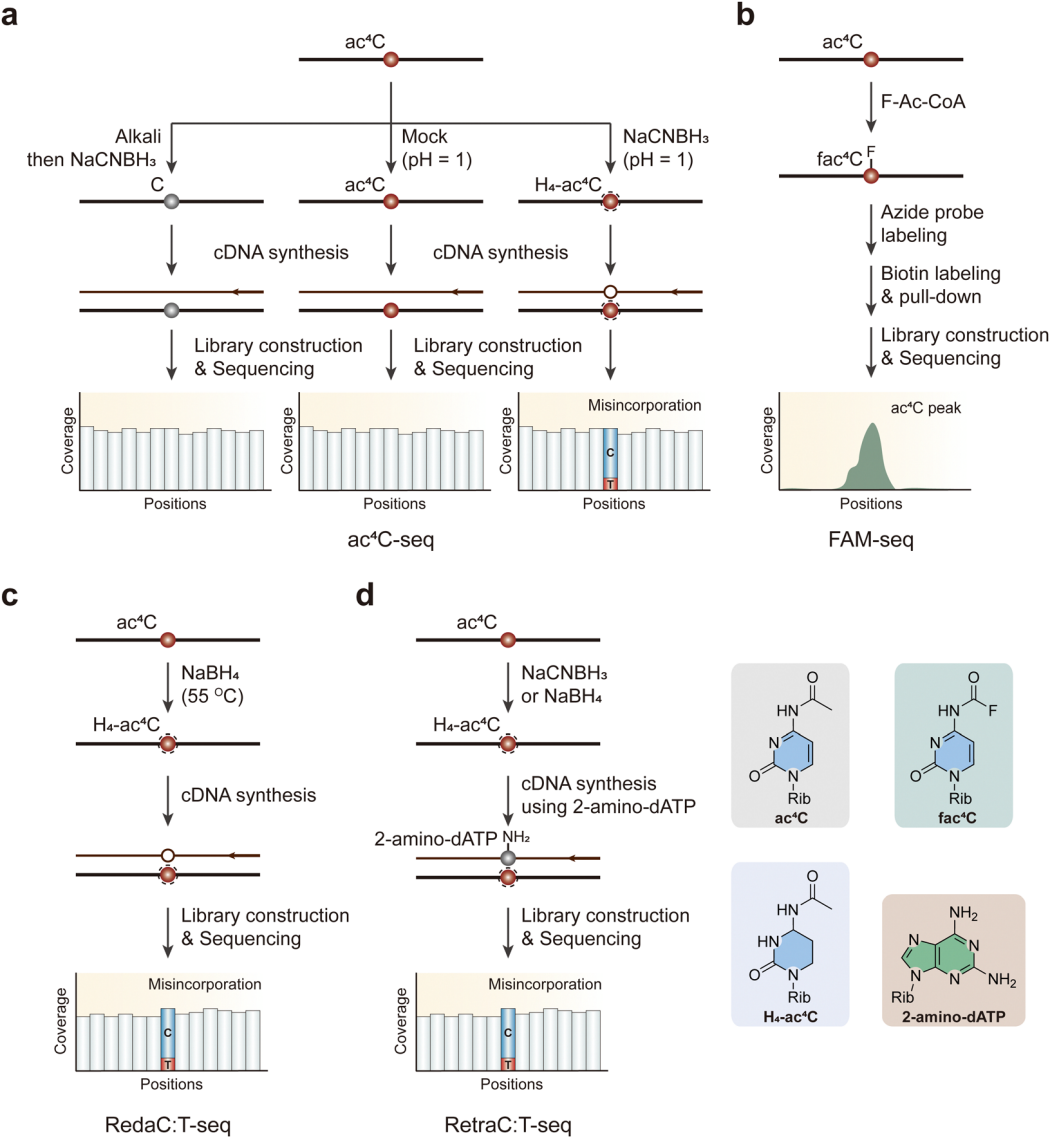
Overview of sequencing methods for the detection of ac^4^C modifications. (a) ac^4^C-seq. (b) FAM-seq. (c) RedaC:T-seq. (d) RetraC:T-seq. Rib, ribose. H_4_-ac^4^C, tetrahydro-*N*^4^-acetylcytidine.

It has been noticed that ac^4^C does not disrupt traditional base pairing. However, when subjected to treatment with two equivalents of sodium cyanoborohydride (NaCNBH_3_), ac^4^C transforms into a reduced nucleobase, tetrahydro-*N*^4^-acetylcytidine (H_4_-ac^4^C), which can be misread as a U during reverse transcription, causing C to T mutations at ac^4^C sites (Fig. 8a).^119^ While several other modified nucleobases (such as m^7^G, dihydropyridine, and *N*^*3*^-methylcytidine) are also susceptible to reduction by hydride donors, the hydrolytic lability of ac^4^C can be exploited to chemically deacetylate RNA for control experiments.^120^ In ac^4^C-seq, one experimental sample is treated with NaCNBH_3_ under acidic conditions (reduction), while two control samples are subjected to acidic conditions without a reducing agent (mock-treated) and deacetylation followed by NaCNBH_3_ treatment under alkali situation (deacetylated and reduction-treated).^119^ Employing ac^4^C-seq allows for the detection of ac^4^C modifications distributed within the transcriptome at single-nucleotide resolution. Nevertheless, this method does demand a higher sample input, and low depth continues to be a significant factor limiting the detection of mRNA acetylation in certain circumstances.

An antibody-free FAM-seq method utilizes antibody-free fluorine-assisted metabolic sequencing to detect ac^4^C within RNA (Fig. 8b).^121^ The acetyl group donor, acetyl coenzyme A (Ac-CoA), serves as the substrate for the ac^4^C methyltransferase NAT10. This enzyme transfers an acetyl group to the *N*^4^-position of cytidines, marking the site of acetylation. To pinpoint cytidine acetylation throughout the transcriptome, researchers fed cells with sodium fluoroacetate. This pro-metabolite compound can be ligated to CoA by acetyl-CoA synthetase within the cell to produce F-Ac-CoA. Subsequently, the acetyltransferase NAT10 transfers the fluoroacetate group from F-Ac-CoA to the *N*^4^ position of a target cytidine in RNA. Previous research has evidenced that fluoroacetamide can be effectively converted to biotin or fluorophore tags through the fluorine-thiol displacement reaction (FTDR) with high selectivity and yield.^122^ Capitalizing on this, the researchers employed an azide probe containing a benzenethiol structure to react with *N*^4^-fluoroacetylcytidine (fac^4^C). Following a click reaction with dibenzocyclooctyne-biotin, the biotin-labeled modified RNAs were enriched for library construction. This innovative approach allows for a more precise identification and analysis of ac^4^C modifications in the RNA, contributing to our understanding of their role in various cellular processes. However, adding sodium fluoroacetate to cell cultures may lead to inevitable false signals in sequencing data.

Avoiding the side effects caused by metabolic labeling, RedaC:T-seq is an advanced sequencing method designed to identify ac^4^C in RNA with high precision.^123^ This technique involves chemically reducing ac^4^C to tetrahydro-ac^4^C using NaBH_4_, which induces C-to-T mismatches during reverse transcription (Fig. 8c). These mismatches are detectable by sequencing and serve as markers for ac^4^C sites. Key features of RedaC:T-seq include its high sensitivity and specificity, achieved by comparing treated samples to untreated and NAT10-knockout controls, ensuring accurate identification of ac^4^C sites. The comprehensive coverage allows for detailed mapping of ac^4^C across various RNA regions even in low-abundance transcripts. However, some researchers have wondered whether the ac^4^C sites provided are not reproducible because of irreproducibility of the mismatch pattern, technical biases and low complexity reads in the sequencing data.^124^ Thus, avoiding the inefficient reduction reaction and low mismatch rate, which results in the inability of RedaC:T-seq to detect ac^4^C modification on mRNAs, researchers have developed an improved method called RetraC:T-seq (Fig. 8d).^125^ This method utilizes NaBH_4_ or NaCNBH_3_ to reduce ac^4^C to tetrahydro-ac^4^C, which leads to C-to-T mismatches during cDNA synthesis with modified dNTPs, such as 2-NH_2_-dATP. These mismatches are then detected *via* sequencing, allowing for precise mapping of ac^4^C sites. This technique offers improved sensitivity and specificity over previous methods, facilitating better understanding of its role in RNA biology.

### 3.6 m^7^G detection methods

m^7^G, a positively charged, crucial modification mainly found at the cap of eukaryotic mRNA, participates in regulating mRNA export, translation, and splicing.^75,126–128^ m^7^G also appears internally within tRNA, rRNA, and eukaryotic mRNA.^75,129–136^ The primary techniques for detecting internal m^7^G in mRNA include antibody-based and chemical-assisted sequencing methods. The m^7^G-MeRIP-seq method, which employs an m^7^G-specific antibody, has successfully identified over 3000 internal m^7^G peaks in mammalian cell lines.^25^ Nevertheless, this approach, dependent entirely on an anti-m^7^G antibody to enrich RNA fragments containing m^7^G, is hindered by several drawbacks, such as a lack of single-base resolution, low detection sensitivity, and high background noise. To address these issues, chemical-assisted sequencing methods that leverage the unique chemical reactivity of m^7^G have been developed, enabling the detection of m^7^G methylome distribution features in human cells at base resolution.

**Fig. 9:**
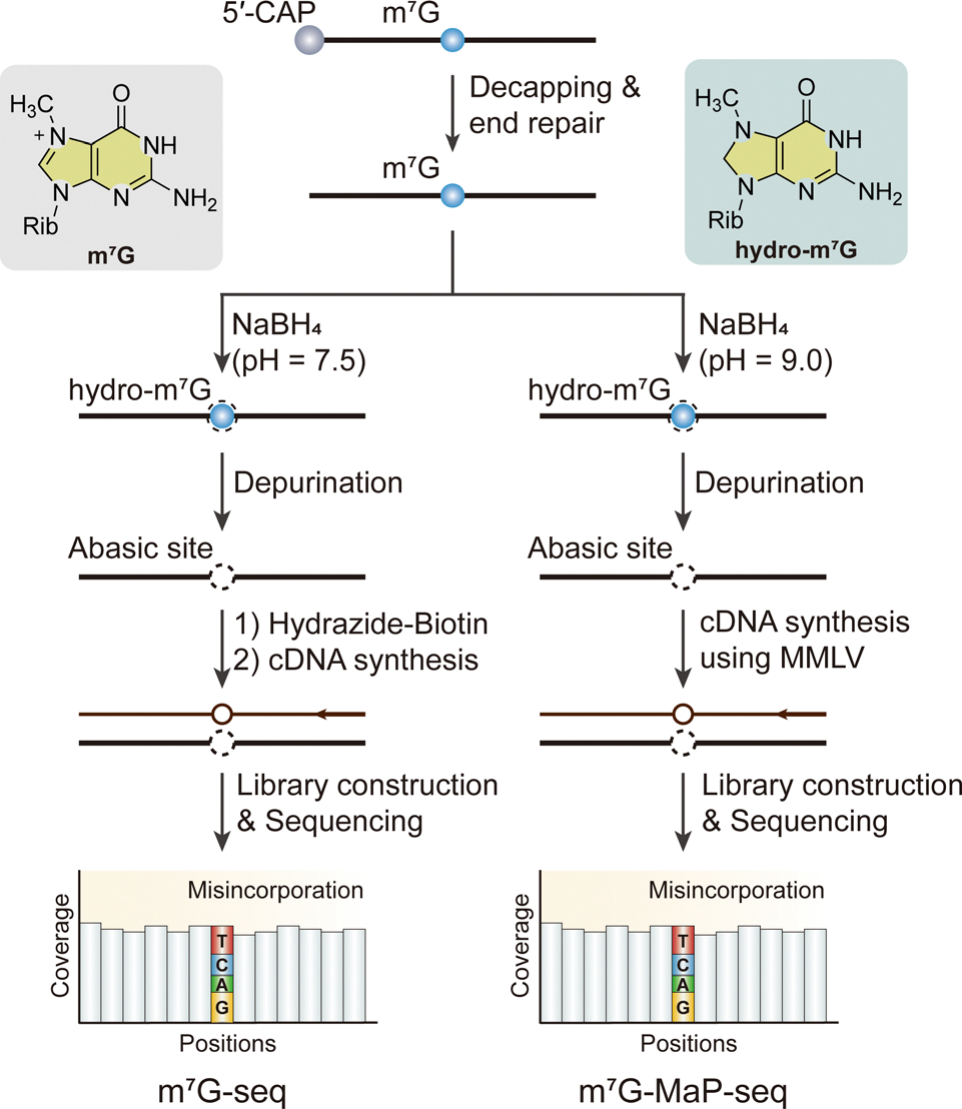
Overview of sequencing methods for the detection of internal m^7^G modifications. Rib, ribose. MMLV, moloney murine leukemia virus reverse transcriptase.

To achieve base resolution mapping of m^7^G with an orthogonal approach, several chemical-assisted sequencing methods, which are referred to as m^7^G-seq, borohydride reduction sequencing (BoRed-seq), mutational profiling sequencing (m^7^G-MaP-seq), tRNA reduction and cleavage sequencing (TRAC-seq) and m^7^G-seq with stoichiometry information (m^7^G-quant-seq) were developed by using the unique chemical reactivity of m^7^G in a reduction-induced depurination reaction (Fig. 9).^25,53,137–139^ However, m^7^G-seq and m^7^G-MaP-seq are two of the methods capable of mapping m^7^G sites on mRNAs, as demonstrated by their results, particularly after incorporating a decapping step into the protocol to generate the necessary substrate for sequencing.^25,137^ In m^7^G-seq, the positive charge on the five-membered ring makes m^7^G particularly susceptible to NaBH_4_-mediated reduction, which eliminates the aromaticity of the five-membered ring attached to the ribose without affecting unmodified G. Reduced m^7^G forms an apurinic/apyrimidinic site (AP site), also known as an abasic site, after heating in an acidic solution, generating an RNA abasic site that can be captured by biotin-ligated hydrazide in a one-pot reaction, resulting in biotinylated RNA. The biotinylated sites are predominantly mutated to T, as well as other bases, during HIV-1 reverse transcriptase (RT)-mediated reverse transcription, enabling the detection of m^7^G sites at single-base resolution.^25^ Similarly, BoRed-seq, TRAC-seq and m^7^G-quant-seq also employ the NaBH_4_- or KBH_4_-mediated reduction process, followed by depurination under mild conditions to generate abasic sites.^25,53^ In TRAC-seq, an additional aniline treatment is employed to facilitate the cleavage of the RNA backbone specifically at m^7^G-modified sites. In m^7^G-MaP-seq, NaBH_4_-meidated RNA abasic sites were misincorporated with moloney murine leukemia virus reverse transcriptase (MMLV) reverse transcriptase to record the positions of m^7^G modifications during reverse transcription.^137^ All these chemical-assisted high throughput sequencing methods for m^7^G could provide precise location of m^7^G at the nucleotide level in various RNA types, while the treatment of NaBH_4_ might affect other types of RNA modification and a high sequencing depth is needed because of RNA degradation.

### 3.7 Ψ detection methods

Ψ is one of the most abundant RNA modifications in eukaryotes, earning it the title of the “fifth nucleoside”.^112^ In yeast, Ψ is present at many positions in tRNAs, at 46 positions across the four rRNAs (25S, 18S, 5.8S, and 5S), and at six positions in snRNA U1, U2, and U5.^140–146^ Although base pairing of Ψ is similar to uridine, isomerization allows the potential formation of an extra hydrogen bond.^147^ This additional bond could lend greater structural stability to RNA molecules, underlining the functional importance of Ψ beyond mere structural decoration. Moreover, Ψ has also been identified in mRNA, highlighting its broader role in the RNA world beyond its traditional confines within rRNA and tRNA. Mass spectrometry studies have quantified the ratio of Ψ to uridine (Ψ/U) in human cell lines, finding it to be on the order of approximately 0.2–0.6%, which is comparable to the ratio of m^6^A to adenosine (m^6^A/A) in these cells.^148^ This similarity in abundance with m^6^A suggests that Ψ might also have important functions in RNA biology.

**Fig. 10:**
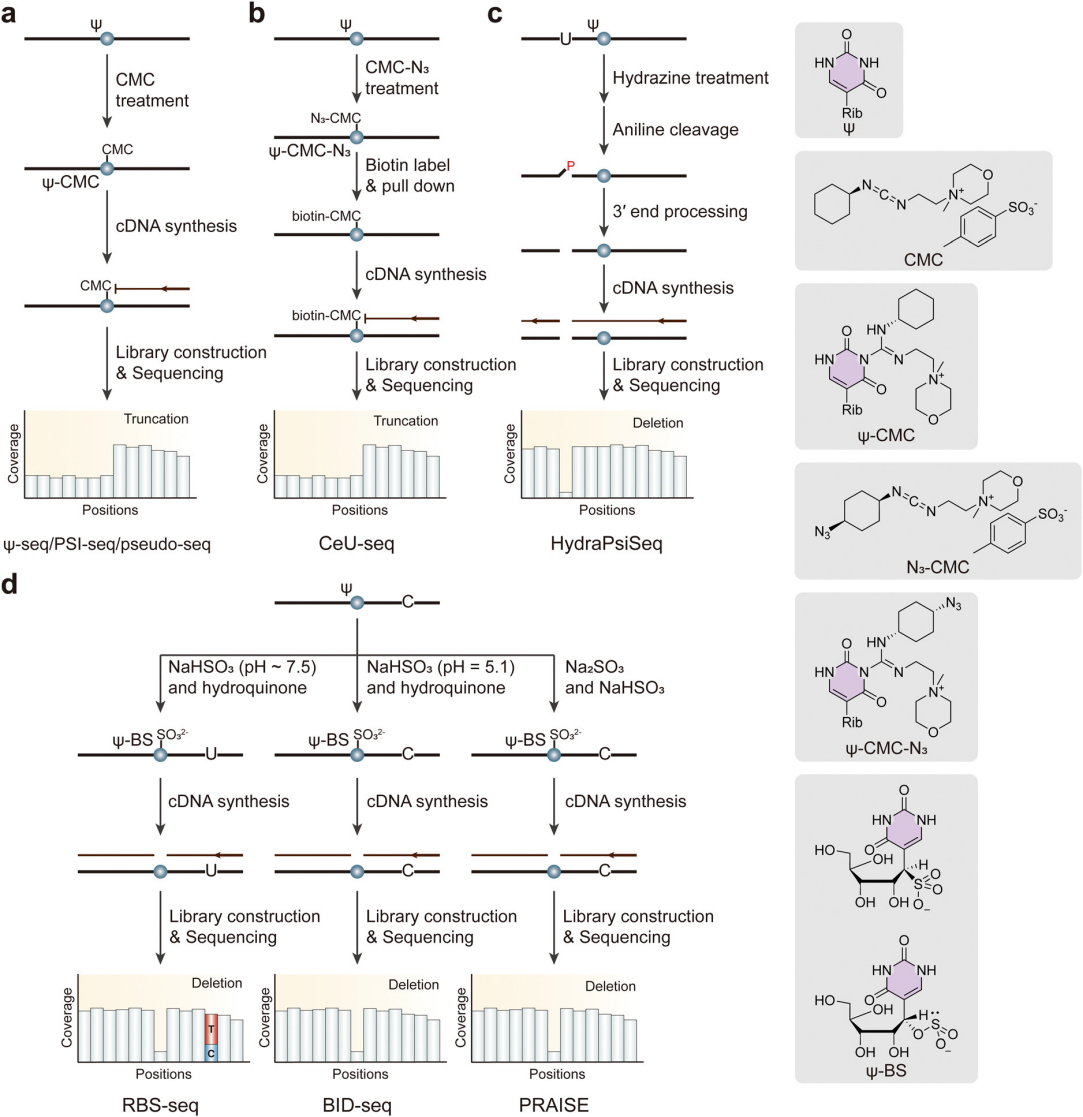
Overview of sequencing methods for the detection of Ψ modifications. (a) Ψ-seq, PSI-seq and pseudo-seq. (b) CeU-seq. (c) HydraPsiSeq. (d) RBS-seq, BID-seq and PRAISE. CMC, *N*-cyclohexyl-*N*′-(4-methylmorpholinium) ethylcarbodiimide. Rib, ribose.

The challenge of Ψ detection in RNA sequences stems from the fact that Ψ is mass-silent and indistinguishable from regular uridine bases during reverse transcription. This difficulty has led to the development of chemical-assisted sequencing methods that rely on the specificity of *N*-cyclohexyl-*N*′-(4-methylmorpholinium)ethylcarbodiimide (CMC) for labeling and distinguishing Ψ from U.^149^ The mechanism of CMC is based on its covalent binding to the *N*^3^ position of U, G, and Ψ residues, resulting in the formation of CMC-U, CMC-G, and CMC-Ψ adducts, respectively. Upon alkaline treatment, the unique chemical stability of the CMC-Ψ adduct under these conditions means it remains intact while the CMC moieties linked to U and G are removed. This stability is harnessed in sequencing methodologies; the presence of a CMC-Ψ adduct causes reverse transcription to terminate, thus facilitating the detection of Ψ at single-base resolution. Using this strategy, three CMC-based profiling methods, including pseudo-seq, Ψ-seq, and PSI-seq, have been successful in mapping Ψ modifications at single-base resolution, particularly in yeast and human mRNA (Fig. 10a).^57,150,151^ However, existing profiling methods do not pre-enrich Ψ-containing RNAs, potentially missing low-abundance pseudouridylation events. To address this, CeU-seq employs a CMC derivative, N_3_-CMC, which forms Ψ-CMC-N_3_ adducts that can be further labeled with biotin for pull-down assays, allowing pre-enrichment of Ψ-containing RNA fragments (Fig. 10b).^148^ Remarkably, CeU-seq has successfully identified a significant number of Ψ sites across various samples, including 1889 sites in human mRNA from HEK293T cells, 1543 sites in mouse liver, and 1741 sites in mouse brain. Furthermore, the detection capabilities of Ψ-CMC-induced mutation/deletion patterns can also be combined with highly sensitive qPCR analysis,^152^ allowing for the detection of locus-specific Ψ modifications across different RNA types, thus broadening the scope and application of pseudouridylation detection in RNA biology.

Since CMC-based profiling methods are prone to variation, making this approach only semi-quantitative, the lack of a quantitative method hinders our ability to comprehensively understand the prevalence of pseudouridylation in the transcriptome and to evaluate its dynamics. To address this issue, a novel quantitative Ψ mapping technique, HydraPsiSeq, was developed. This method relies on specific protection from hydrazine/aniline cleavage of Ψ (Fig. 10c).^153^ In principle, hydrazine cleaves uridine residues, forming abasic sites that are then treated with aniline to fragment the RNA strands. Since Ψ is unaffected by hydrazine, intact RNA fragments are retained. Ψ sites can be precisely located and quantitatively analyzed by comparing them to the reference genome. Although this strategy does not allow enrichment, HydraPsiSeq provides a systematic approach for mapping and accurately quantifying pseudouridines in RNAs, with potential applications in disease, development, and stress response.

Additionally, Ψ can undergo irreversible labeling through a bisulfite reaction combined with hydroquinone,^154^ leading to the formation of a ribose ring-opening adduct. This Ψ-bisulfite adduct was later found to induce reverse transcriptase bypass, allowing its detection as 1–2 nucleotide deletion signatures during sequencing (Fig. 10d).^110^ However, traditional bisulfite conditions, such as RBS-seq, while capable of detecting m^1^A simultaneously, suffer from limited labeling efficiency, which can result in incomplete conversion and an increased deletion rate. This limitation restricts the accuracy of methylation detection and can obscure subtle epigenetic modifications. To this end, two independent studies introduced approaches for the absolute quantification of transcriptome-wide Ψ, namely BID-seq and PRAISE.^155,156^ BID-seq employs an adjusted pH with sodium hydroxide (NaOH), while PRAISE enhances effective ion concentrations during the bisulfite reaction without adding hydroquinone. Both methods inhibit C-to-T conversion and significantly improve reaction efficiency towards Ψ. In the context of mRNA modification, BID-seq and PRAISE identified thousands of Ψ sites along with their modification stoichiometry, highlighting the absolute quantitative capability of the optimized bisulfite chemistry.

### 3.8 I detection methods

Recent studies on inosine and A-to-I (adenosine-to-inosine) RNA editing reveal that it's a common feature across various transcripts, indicating its significant biological function.^157^ This A-to-I editing is enzymatically facilitated by the family of adenosine deaminases acting on RNA (ADAR) enzymes.^157^ ADAR-mediated A-to-I base editing, particularly in dsRNA regions, results in the conversion of A to I, which is then interpreted as G during translation. To achieve accurate detection of inosine modifications on a transcriptome-wide level, a variety of high-throughput sequencing methods have been established, each employing unique strategies such as direct sequencing, chemical-assisted sequencing, and enzyme-based enrichment techniques.

The most conventional method to identify A-to-I editing sites is direct sequencing by comparing cDNA sequences with their corresponding genomic DNA sequences. This approach relies on the reverse transcription step, where inosines are read as guanosines. Thus, the appearance of A-to-G mismatches between the cDNA and the genomic sequence is indicative of A-to-I editing. However, this method can be limited by the difficulty in distinguishing true editing events from sequencing errors or PCR artifacts, particularly in regions with high noise or pseudogene regions.

**Fig. 11:**
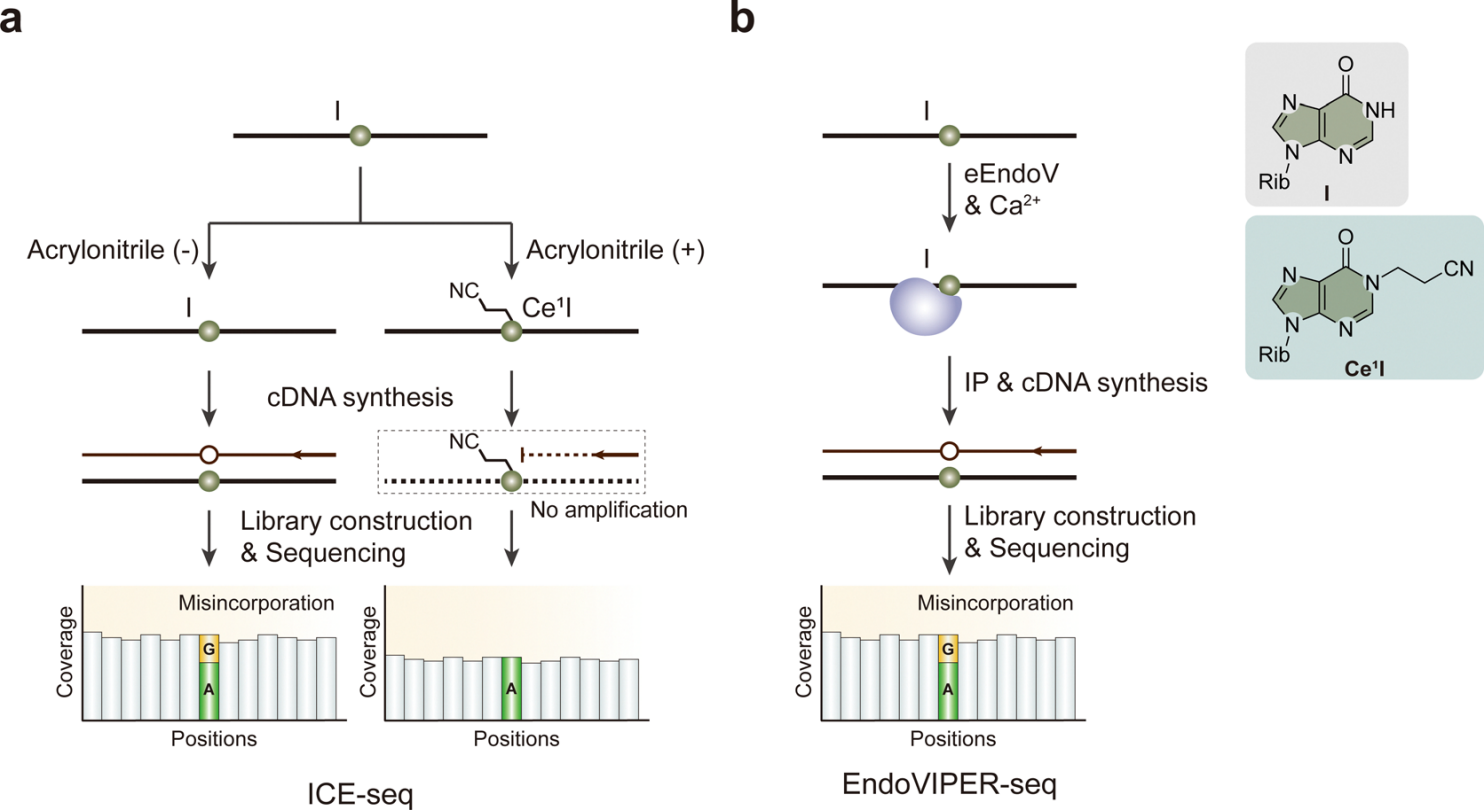
Overview of sequencing methods for the detection of I modifications. (a) ICE-seq. (b) EndoVIPER-seq. eEndoV, *Escherichia coli* endonuclease V. Rib, ribose.

To overcome the limitations of direct sequencing, chemical-assisted approaches like inosine chemical erasing (ICE) have been developed (Fig. 11a).^158^ Combined with high-throughput sequencing, ICE-seq is based on the cyanoethylation of inosines, which blocks reverse transcription at modified sites, thereby allowing direct identification of inosines in sequencing reads. This method is highly specific and may not require genomic DNA as a reference, making it a reliable technique for detecting editing events without the confused effects of SNP (single nucleotide polymorphism) or sequencing errors.

Another important method for identifying inosine modifications involves enzyme-based enrichment, called endonuclease V inosine precipitation enrichment sequencing (EndoVIPER-seq) (Fig. 11b).^159^ It uses specific endonuclease eEndoV (*Escherichia coli* endonuclease V) to recognize fragmented inosine-containing RNA, enriching for edited regions before sequencing. This approach can improve the sensitivity of inosine detection, particularly in samples with low editing frequencies or in non-repetitive regions where editing is not abundant.

These diverse sequencing methods have collectively advanced our understanding of A-to-I RNA editing, revealing its widespread occurrence across the transcriptome and its involvement in numerous biological processes. As new techniques continue to be developed, the resolution and accuracy of inosine detection are expected to improve, providing deeper insights into the function of inosine across the transcriptome.

### 3.9 Nm detection methods

The post-transcriptional modification 2′-*O*-methylation (Nm), found on the ribose of all four ribonucleosides, is a significant RNA modification commonly recognized in noncoding RNAs. This includes the 5′ RNA cap in viruses and higher eukaryotes, as well as within small nucleolar RNA (snoRNA) and rRNA.

**Fig. 12:**
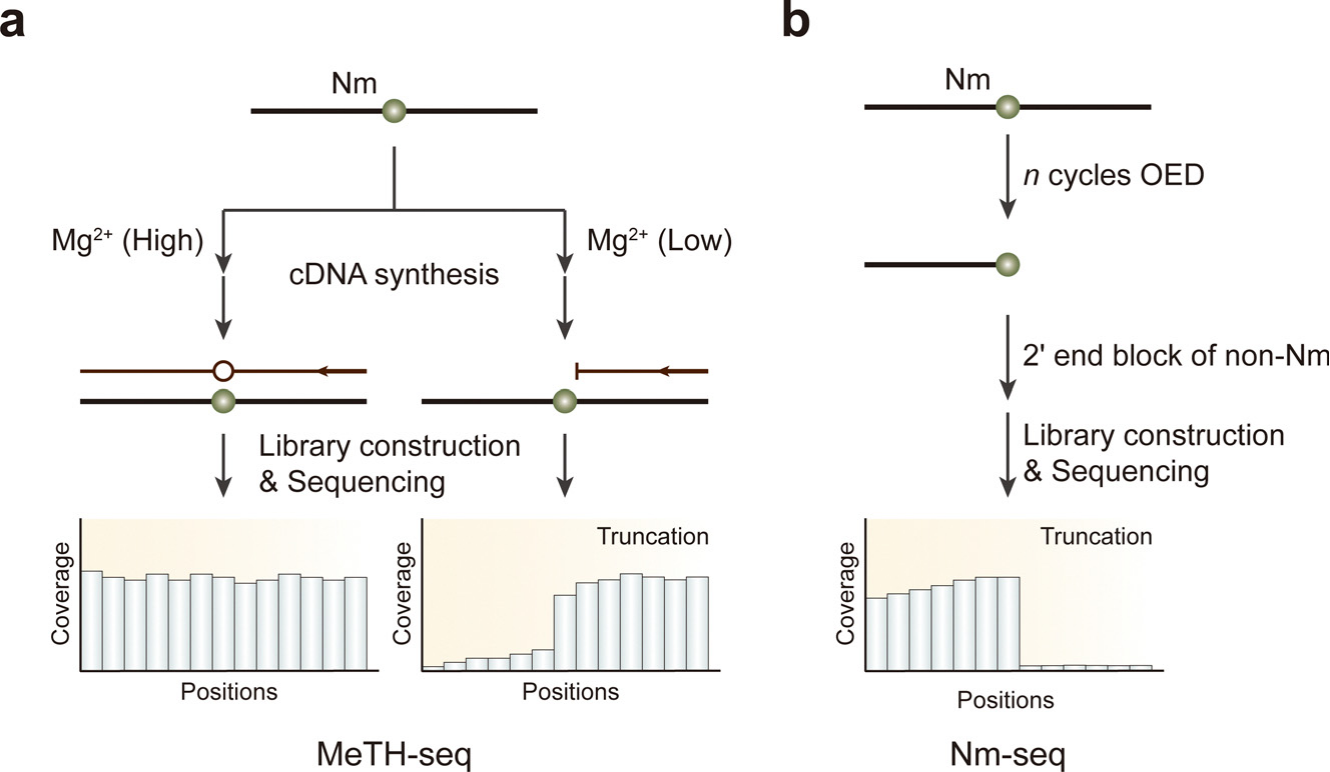
Overview of sequencing methods for the detection of Nm modifications. (a) MeTH-seq. (b) Nm-seq. OED, oxidation-elimination-dephosphorylation.

Recent advancements have led to the development of techniques for transcriptome-wide mapping of Nm using high-throughput sequencing, capable of confirming established Nm sites on abundant rRNA. RiboMeth-Seq employs alkaline RNA cleavage followed by high-throughput sequencing; because 2′-*O*-methylation protects nucleotides from alkaline fragmentation, modified residues are excluded from the RNA library.^160,161^ Similarly, RibOxi-seq also utilizes alkaline RNA cleavage but is enriched for methylated fragments by preventing the ligation of unmethylated fragments to adaptors.^162^ Other methods that exploit the steric properties of Nm include 2OMe-seq and MeTH-seq, which sequence a cDNA library prepared through reverse transcription under restrictive conditions, such as low dNTP or low magnesium concentrations, causing reverse transcription to terminate at Nm sites (Fig. 12a).^163,164^ However, only MeTH-seq is used to map Nm sites on mRNAs.

Although these approaches effectively detect Nm modifications on abundant RNAs, they encounter challenges when applied to less abundant RNAs, such as mRNA. These methods often lack stoichiometric information and can lead to RNA sample degradation due to chemical treatments. To achieve single-nucleotide precision profiling in mRNA species, Nm-seq was developed to map thousands of Nm sites in human mRNA (Fig. 12b).^165^ Nm-seq employs multiple rounds of oxidation-elimination-dephosphorylation (OED) to iteratively remove 2′-unmodified nucleotides from the 3′ end of fragmented RNA. 2′-*O*-Methylated nucleotides resist OED, resulting in their enrichment at the 3′ end of the fragments. After several rounds of OED, a final oxidation-elimination reaction is performed without dephosphorylation, creating unligatable 3′ monophosphate ends on fragments ending with unmodified nucleotides. In contrast, 3′ adaptors are ligated onto fragments with 2′-*O*-methylated ends that retain a ligatable 3′ OH. Consequently, the 3′ end of each RNA fragment corresponds to the 2′-*O*-methylated nucleotide, which is then mapped using high-throughput sequencing.

## 4. Conclusions and outlook

In the last decade, the field of RNA epigenetics has advanced remarkably, starting with the discovery of the reversible m^6^A modification.^166^ Initially, detection methods required large amounts of material and were costly, but newer techniques now provide single-nucleotide resolution with lower input requirements and costs. This progress has been driven by the development of reactivity-based sequencing methods, which have significantly enhanced our ability to profile RNA modifications with high precision and sensitivity.

These methods are notable for their high sensitivity and specificity, allowing for the detection of low-abundance modifications and offering comprehensive coverage of RNA modification landscapes. Their ability to provide single-base resolution is essential for understanding the precise roles of these modifications in RNA biology. Additionally, these techniques are antibody-free, which reduces the risk of cross-reactivity and false positives, enhancing the reliability of the results.

Despite these advancements, challenges remain. The complexity of these procedures and the need for specialized equipment and expertise can limit accessibility. High sequencing depth requirements can be resource-intensive, and potential technical biases necessitate careful experimental design and data analysis. Some modifications, such as hm^5^C, are still challenging to precisely detect with current methods. Continuous refinement and the development of orthogonal validation methods are essential for improving accuracy and expanding applicability.

Looking ahead, future efforts should focus on developing absolute quantitative methods, single-cell level analyses, and time-resolved studies of RNA modification dynamics. Nanopore sequencing, which has shown potential for simultaneous detection of multiple modifications at single-base resolution, requires improvements in cost and accuracy.^167–169^ Combining enzyme- and chemical-assisted methods could enhance detection signals.

Moreover, many RNA modifications with significant biological functions remain undetected by current high-throughput methods. Optimizing existing technologies could help identify these modifications. The discovery of new modifications and sequencing techniques will likely accelerate the use of RNA modifications as biomarkers for disease diagnosis and treatment.^170^

In summary, advancing RNA modification detection technologies will likely focus on increasing sensitivity and specificity, reducing complexity, and minimizing technical biases. These improvements will broaden the application of these methods in diverse biological contexts, including gene regulation, cellular processes, and disease mechanisms. Understanding RNA modifications and their roles will be crucial for developing targeted therapies, underscoring the importance of continued research in this field. Because of the complex roles of RNA modifications, this requires advanced mapping and quantification methods as existing methods can hardly identify many kinds of modifications across all RNA types simultaneously, which might by an important point for medical diagnosis of diseases. This is further complicated by the lack of type-specific modification patterns and varying abundance across RNAs. Understanding RNA modifications and their roles will be crucial for developing targeted therapies, underscoring the importance of continued research in this field, such as pseudouridine and *N*^1^-methylpseudouridine (m^1^Ψ) in mRNA vaccine immunogenicity and effective half-life.^171–173^ In a larger sense, it is clear that the function of RNA modifications makes them an excellent therapeutic target for further investigation.

## Data availability

No primary research results, software or code have been included and no new data were generated or analysed as part of this review.

## Conflicts of interest

There are no conflicts to declare.

## Supplementary Material

CB-OLF-D4CB00215F-s001
